# Mechanistic insights into the role of plant polyphenols and their nano-formulations in the management of depression

**DOI:** 10.3389/fphar.2022.1046599

**Published:** 2022-11-07

**Authors:** Atul Kabra, Ruchika Garg, James Brimson, Jelena Živković, Saud Almawash, Muhammad Ayaz, Asif Nawaz, Syed Shams Ul Hassan, Simona Bungau

**Affiliations:** ^1^ University Institute of Pharma Sciences, Chandigarh University, Mohali, Punjab, India; ^2^ University School of Pharmaceutical Sciences, Rayat Bhara University, Mohali, Punjab, India; ^3^ Natural Products for Neuroprotection and Anti-Ageing Research Unit, Chulalongkorn University, Bangkok, Thailand; ^4^ Department for Pharmaceutical Research and Development, Institute for Medicinal Plants Research “Dr. Josif Pančić”, Belgrade, Serbia; ^5^ Department of Pharmaceutical Sciences, College of Pharmacy, Shaqra University, Shaqra, Saudi Arabia; ^6^ Department of Pharmacy, Faculty of Biological Sciences, University of Malakand, Chakdara, Pakistan; ^7^ Shanghai Key Laboratory for Molecular Engineering of Chiral Drugs, School of Pharmacy, Shanghai Jiao Tong University, Shanghai, China; ^8^ Department of Natural Product Chemistry, School of Pharmacy, Shanghai Jiao Tong University, Shanghai, China; ^9^ Department of Pharmacy, Faculty of Medicine and Pharmacy, University of Oradea, Oradea, Romania

**Keywords:** antidepressants, polyphenols, natural products, depression, herbal medicine

## Abstract

Depression is a condition characterized by low mood and an aversion to activity, that causes behavioral problems, poor quality of life and limits daily life activities. It is considered as the fourth leading cause of disability worldwide. Selective Serotonin Reuptake Inhibitors (SSRIs) Monoamine Oxidase (MAO) inhibitors, Tricyclic Antidepressants (TCAs), and atypical antidepressants are some of the conventional medications used to treat depression. However, only about half of patients with major depressive disorder (MDD) respond effectively to first-line antidepressant therapy. Additionally, there are a number of drawbacks to standard antidepressants, such as anti-cholinergic side effects, drug-drug interactions, and food-drug interactions, which prompts researchers to look at alternative approaches to the treatment of depression. Medicinal plants and their metabolites are extensively tested for their efficacy against depression. Electronic databases such as Google scholar, Science Direct, SciFinder and PubMed were used to search relevant literature on the role of polyphenols in depression. Plants-derived Polyphenols represent a major class of compounds extensively distributed in plants. Number of polyphenols have demonstrated antidepressant activity, among which berberine, piperine, curcumin, naringenin, ascorbic acid and ginsenosides are extensively evaluated. The medicinal plants and their derived compounds mediated synthesized green nanoparticles have also exhibited considerable efficacy in the management of depression. The therapeutic effects of these phytochemicals is mediated *via* differentiation and inhibition of neuronal cell apoptosis, promotion of neuronal cell survival and modulation of key neurotransmitters. The aim of this study is to review compressively the chemical, pharmacological and neurological evidence showing the potential of polyphenols in depression.

## Introduction

Depression is a condition characterized by low mood and an aversion to activity. Low mood is a regular and expected part of the human condition, particularly when grieving loved ones. However, major depression disorder (MDD) is defined as a constant state of low mood that lasts for at least 2 weeks (Otte et al., 2016). Persistent depressive disorder (dysthymia), which may be less severe than MDD, lasts much longer (typically two or more years) (Baldwin et al., 1995). Other forms of depression include perinatal depression (occurring during or shortly after pregnancy in women) and seasonal affective disorder (occurring typically in the autumn or winter, with symptoms fading in the spring and summer). Depression affects a patient’s thoughts, mood, behavior, sense of well-being, and motivation. Patients may also have feelings of hopelessness, dejection, and possibly thoughts of suicide (de Zwart et al., 2019). Depression is thought to affect 5% of the world’s population (Institute of Health Metrics and Evaluation, 2021), and suicide rates in the United States of America rose alarmingly between 1999 and 2016 (Stone et al., 2018). According to the 2017 Global Burden of Disease (GBD) study the global incidence of depression cases between 1990 and 2017 has increased by 49.86% (Liu et al., 2020). The point prevalence rate of depressive symptoms was 34% from 2001 to 2020 globally, and for MDD was 8%. Among adolescents, the point prevalence of elevated depressive symptoms increased from 24% (between 2001 and 2010) to 37% (between 2011 and 2020) (Shorey et al., 2022). Furthermore, the World Health Organization lists suicide as the fourth leading cause of death in 15–29-year-olds worldwide (World Health Organization, 2021). Despite several clinically effective drugs for treating depression, people in more than 75% of middle and low-income counties do not receive treatment (Evans-Lacko et al., 2018; Ahsan et al., 2022).

Number of antidepressant agents such as SSRIs, MAO inhibitors and TCAs etc are commercially available which are in practice for the treatment of depression. The acute or chronic use of these agents may result in serious effects such as adverse drug reactions, drug-drug interactions or drug-food interactions (Pathak et al., 2013). These factors prompt the researchers to develop new antidepressant agents having minimal side effects and better efficacy. Medicinal plants and their metabolites are extensively tested for their efficacy against depression. Number of polyphenols have demonstrated antidepressant activity, among which berberine, piperine, curcumin, naringenin, ascorbic acid and ginsenosides are extensively evaluated. The therapeutic effects of these phytochemicals is mediated *via* differentiation and inhibition of neuronal cell apoptosis, promotion of neuronal cell survival and modulation of key neurotransmitters (Dhir, 2017). The present review aims to discuss compressively the antidepressant potential of polyphenols, including their mechanisms of action.

## The monoamine hypothesis

**FIGURE 1:**
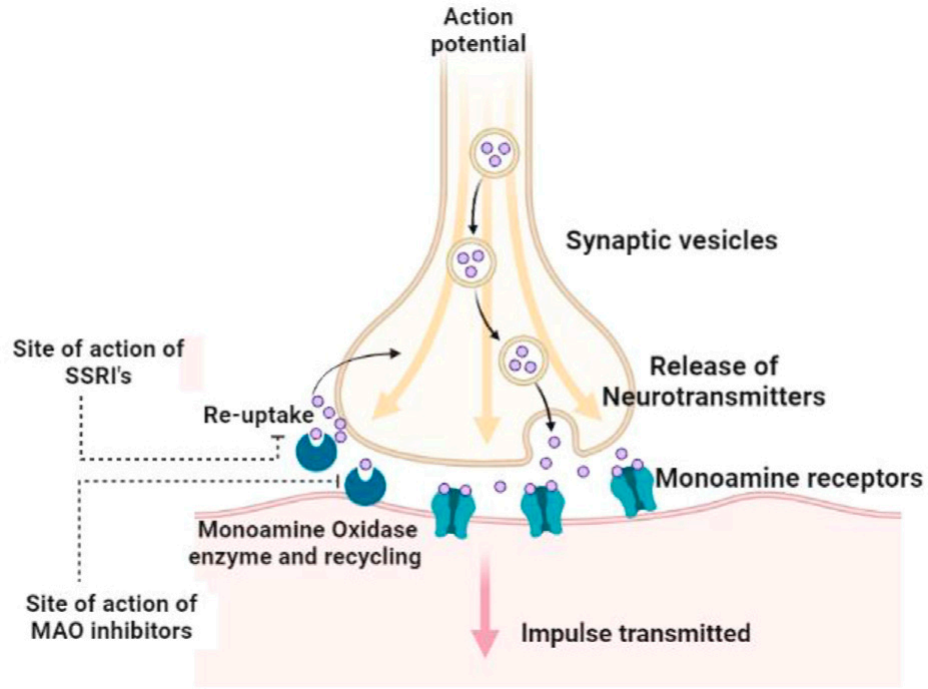
The monoamine (MAO) theory of depression; antidepressant drugs increase monoamine concentrations in the synaptic cleft leading to further signaling and the postsynaptic neuron.

The monoamine (catecholamine) hypothesis of depression (Figure 1) was proposed in early 1965's (Schildkraut, 1995) based on pharmacological evidence from drugs. For instance, reserpine and tetrabenazine deplete and inactivate monoamines intracellularly thus causing depression (Ruhé et al., 2007). On the contrary, amphetamine causes an increase in the synaptic norepinephrine levels and thus exhibit stimulant properties. Likewise, the monoaminoxidase (MAO) inhibitors prevent the breakdown of the monoamines within the synaptic cleft, giving rise to apparent antidepressant effects. Finally, the tricyclic class of antidepressants blocks serotonin and norepinephrine reuptake by serotonin and norepinephrine reuptake transporters (SERT and NET) in the synaptic cleft (Gillman, 2007). The hypothesis existed in the background until the late 1970s with the development of the first generation of selective serotonin reuptake inhibitors (SSRIs) (Spinks and Spinks, 2002) using rational design to improve the specificity of a drug to its intended target (Carlsson, 1999). Several antihistamines were known to have some antidepressant effects, which led to the development of zimelidine from the antihistamine drug bromopheniramine (Fagius et al., 1985), and later fluoxetine from the antihistaminic drug diphenhydramine (Wong et al., 1995). Fluoxetine (marketed as Prozac or Sarafem) was approved by FDA in the USA for the management of depression in 1987 and has subsequently become a prototype for the next generation of SSRI antidepressants. The development of SSRIs has continued, and there are now several on the market including citalopram (Celexa), escitalopram (Lexapro), fluvoxamine (Luvox), paroxetine (Paxil), and sertraline (Zoloft).

It is proposed that SSRIs cause short-term increase serotonin levels in the synaptic cleft, thus stimulating more serotonin receptors on the postsynaptic nerve terminal. Subsequently, it augment serotonin signaling in neurons where serotonin is the primary neurotransmitter. The use of selective drugs that target the reuptake of serotonin in the synaptic cleft and thus increase serotonin’s availability appears to confirm the monoamine hypothesis. However, there are still several problems with the hypothesis. While meta analysis studies of SSRIs for patients with MDD have shown statistically significant improvements. Only in cases of severe symptoms, compared to placebo controls, many of these trials showed a high risk of bias, and the benefits were often outweighed by the risk of harmful side effects (Fournier et al., 2010; Jakobsen et al., 2017).

Furthermore, despite seeing an immediate increase in serotonin levels after treatment with SSRIs and the immediate antidepressant-like effects of SSRIs in animal studies, patients treated for depression with SSRIs do not see an immediate improvement in their condition and often requiring 2 weeks before improvements are observed (Edinoff et al., 2021). Additionally, during that time, there may be an apparent worsening of symptoms. With chronic dosing, the highly occupied serotonin receptors on the postsynaptic membrane trigger feedback signaling, resulting in less serotonin at the presynaptic neuron (Siesser et al., 2013). Furthermore, chronic dosing with SSRIs reduces the density of serotonin transporters (SERT) (Benmansour et al., 1999). Therefore, the regulation on serotonin levels in the synapse and the monoamine theory of depression does not adequately explain the pathophysiology of depression.

### The sigma-1 receptor

The sigma-1 receptor (σ1R) is a chaperone protein found at the endoplasmic reticulum (ER) and mitochondrial membrane (MEM) and at the cell membrane. The σ1R is expressed throughout the body, but it is particularly densely expressed in the CNS. Expression of the σ1R appears to act as a cell survival signal, making it a potential target in many diseases, including cancer (Spruce et al., 2004; Brimson et al., 2020a), Alzheimer’s disease (AD) (Prasanth et al., 2021), Parkinson’s disease (PD) (Brimson et al., 2018), Huntington’s disease (HD), multiple sclerosis, amyotrophic lateral sclerosis (ALS) (Brimson et al., 2020b), heart disease, viral infections (Brimson et al., 2021) and depression (Brimson et al., 2020a).

Sigma-1 receptor is a promising therapeutic target in the treatment of neurodegenerative diseases including depression and schizophrenia as it stabilizes the function of several intracellular systems through its role as a chaperone when activated by a variety of ligands with neuroprotective properties. σ Receptor ligands may cause an inhibition of ischemic-induced presynaptic release of excitotoxic amino acids, indicating that σ ligands could also serve as neuroprotective agents (Maurice and Lockhart, 1997). The saffron extracts and crocetin has been reported to have antidepressant effects due to involvement of σ1Rs (Lechtenberg et al., 2008).

The σ1Rs involvement in the pathophysiology of depression is often hotly debated; however, there is plenty of evidence regarding its possible role. Initial interest in the σ1R for depression treatment stems from the observation that most antidepressant drugs have moderate to high affinity for the σ1R (Narita et al., 1996; Brimson et al., 2020b). Furthermore, many antidepressant drugs act as σ1R agonists (with the notable exceptions of sertraline, a σ1R antagonist, and paroxetine which does not affect the σ1R). The order in affinity of the SSRIs for the σ1R is as follows, fluvoxamine > sertraline > fluoxetine > escitalopram > citalopram >> paroxetine. Among these, fluvoxamine, fluoxetine, and escitalopram can potentiate neurite outgrowth in PC-12 cells in a σ1R antagonist sensitive manner.

Furthermore, fluvoxamine improved phencyclidine-induced cognitive deficits in mice, and this could be reversed by the σ1R antagonist NE-100, whereas sertraline (σ1R antagonist) and paroxetine (low affinity for σ1R) had no such effects on phencyclidine treated mice (Albayrak and Hashimoto, 2017). Clinical evidence also suggests a prominent role for the σ1R in the pathophysiology of the σ1R, as when switching patients with psychotic depression from fluvoxamine (SSRI with strong σ1R agonist activity) to sertraline (SSRI and σ1R antagonist), the patient’s symptoms worsened (Kishimoto et al., 2010). This has led to the implication that the σ1R is involved in the beneficial mechanism of fluvoxamine (Hayashi, 2005; Ishikawa and Hashimoto, 2010; Albayrak and Hashimoto, 2017).

Studies using animal models have further provided evidence of the σ1R’s involvement in depression. The σ1R ligands such as PRE-084 (Skuza and Rogóż, 2009), (+)-pentazocine, DTG, SKF-10,047 (Urani et al., 2004) and igmesine (Urani et al., 2001) all dose-dependently reduce immobility time in the forced swim test in an antagonist sensitive fashion. Furthermore, σ1R knockout mice show an apparent depressive-like phenotype in the forced swim test (Sabino et al., 2009). Moreover, mice treated with aortic banding and a high salt diet in a heart failure model showed depressive-like symptoms in the tail suspension test and had reduced expression of the σ1R in the brain (Ito et al., 2012).

Clinical studies have been conducted with σ1R specific ligands igmesine (Roman et al., 1990) and opimarol (structurally similar to imipramine) (Müller et al., 2004; Volz and Stoll, 2004). Igmesine, showed a response rate as high as 83%, although later, larger trials were less conclusive (Pande et al., 1999). However, this trial did appear to have a high response rate in the placebo group, which could have clouded the effects.

## Brain regions and depression

Studies have shown that several regions of the brain are affected in patients suffering from depression. Additionally, the heterogeneity among depressed patients makes it difficult to identify specific brain regions among depressed populations and interpret findings (Graham et al., 2013). However, studies have shown the hippocampus as one such region of the brain with atrophy in MDD (Sheline et al., 1996; Lloyd et al., 2004; Cole et al., 2011). Stress can lead to depression-like symptoms and an excess of cortisol production. The hippocampus is particularly rich in glucocorticoid receptors making it more vulnerable to long-term stress than other regions of the brain, and this is the potential cause of hippocampal atrophy (Campbell and Macqueen, 2004). For instance, σ1R knockout mice show decreased neurogenesis in the hippocampus (Sha et al., 2015) and a depressive-like phenotype. Moreover, SSRIs with σ1R affinity such as fluoxetine can prevent stress-induced atrophy (Luo and Tan, 2001) and even induce further neurogenesis (Santarelli et al., 2003).

Other regions of the brain have been identified as either hyper or hypoactive in cases of MDD. The amygdala, part of the brain involved in emotional processing, is hyperactive in those with MDD. The size of the amygdala appears larger in medicated patients than un-medicated, although there is no noticeable difference between depressed patients and healthy people (Hamilton et al., 2008). The prefrontal cortex (PFC) has been identified as underactive in MDD. The PFR regulates emotional processing; its low activity may be involved in the etiology of depression. Treatment with norepinephrine reuptake inhibitors (SNRIs) has been shown to increase activity in the PFR, whereas SSRIs are amygdaloid-hippocampal regions (Outhred et al., 2013). The different brain regions affected by different antidepressant classes may explain the different efficacies of different drugs between patients.

### The immune system, inflammation, and depression

While it is unlikely that inflammation is the primary cause of the MDD, there is evidence that it plays a role. Approximately 33% of MDD cases show an increase in inflammatory markers compared to healthy controls (Krishnadas and Cavanagh, 2012). Also, patients with inflammatory diseases are at greater risk of MDD (Choi et al., 2019), and treatment with anti-inflammatory drugs can reduce depression symptoms (Köhler et al., 2014). Moreover, treatment with cytokines such as interferon-alpha (IFN- ɑ) (used in treatments for cancer and chronic hepatitis C) can result in an increased risk of depression, with studies suggesting a prevalence of 20%–30% (Sockalingam et al., 2011).

Multiple studies in animals have identified the effects of inflammatory cytokines serotonin turnover in the brain (Dunn and Wang, 1995; Dunn et al., 1999; Anisman et al., 2005). Clinical studies have shown that the SSRI paroxetine can reduce symptoms of depression in patients treated with IFN-ɑ (Musselman et al., 2001; Lotrich et al., 2009). Furthermore, pro-inflammatory cytokines have been shown to affect dopamine production and reuptake in the brain (Morón et al., 2003; Wu et al., 2007). Glutamate is another excitatory neurotransmitter associated with inflammation and neurological disease through excitotoxicity and oxidative stress (Brimson et al., 2018). IL-1β prevents the reuptake of glutamate by glial cells, which leads to increased glutamate in the synapse and causes further NMDA-mediated excitotoxicity. Furthermore, IL-1β induces nitric oxide synthase production and thus an increase in nitric oxide (NO) production, leading to further glutamate release causing further excitotoxicity and oxidative stress (Hu et al., 2000).

### Genetics of depression

The heritability of depression has been identified in twin studies, with estimates suggesting heritability ranged from 22% to 37% (McGue and Christensen, 2003) and as high as 70% in another (Kendler et al., 1993). This strongly suggests a genetic link when it comes to the development of the MDD.

While there may well be a genetic link to many forms of depression, once again, the heterogeneous nature of MDD has made it difficult to identify genes that contribute to increased risk. Two possible candidate genes include the serotonin transporter genes and brain-derived neurotrophic factor (BDNF). The serotonin transporter genes and genes related to its expression and the serotonin receptor 2A (5Ht-2A receptor) have long been candidates due to their proposed role in the monoamine hypothesis of depression (Lohoff and Ferraro, 2010). A 44 base pair repeat polymorphism in the promoter of the serotonin transporter (5-HTTLPR) has been shown, *in vitro*, to influence the expression of the transporter (Lesch et al., 1996). The Val66Met polymorphism in BDNF has been extensively studied in bipolar disorder and MDD. The results for the association between the Val66Met single nucleotide polymorphism (SNP) and other polymorphisms in BDNF and MDD have been mixed. Haplotype analysis of BDNF indicated an association with MDD (Schumacher et al., 2005), whereas studies of the Val66Met SNP have yielded negative results (Surtees et al., 2007).

## Plant polyphenols: Sources and chemistry

Medicinal plants is a rich source of bioactive compounds with diverse pharmacological potentials (Mir et al., 2019; Muhammad et al., 2021a; Muhammad et al., 2021b; Muhammad et al., 2021c). Polyphenols represent greatly varying group of compounds extensively distributed in plants (Ovais et al., 2018b; Zohra et al., 2019). Their chemical structure as well as their composition are significantly influenced by environmental factors and plant species (Ofosu et al., 2020). According to their structure and complexity, they can be classified in two main sub-groups: flavonoids (flavones, flavonols, flavanones, flavanonols, isoflavonoids, flavanols, anthocyanidins and chalcones) and non-flavonoid compounds (phenolic acids, stilbenes, lignans and tannins) (Ayaz et al., 2019) Figure 2.

**FIGURE 2:**
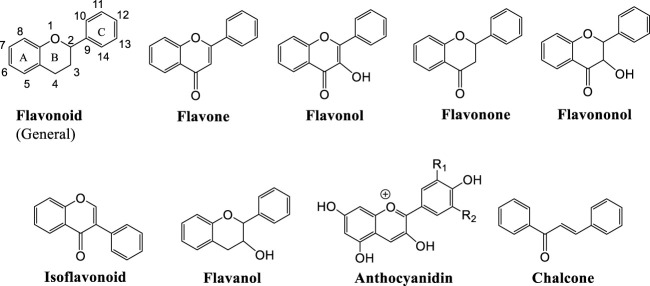
Major classes of flavonoids.

### Flavonoid compounds

Flavonoids constitute a wide group of at least 6000 polyphenolic compounds which differ in their structure but generally, they contain two aromatic rings (A and B rings) linked by a 3-carbon chain making an oxygenated heterocyclic ring (C ring). Based on variations in structure of C ring, as well as functional groups on the rings and the position at which the B ring is connected with the C ring, there are six subclasses of flavonoids, namely flavones, flavonols, flavanones, flavanols, isoflavones and anthocyanidins (Ayaz et al., 2019). Six-member ring linked with the benzene ring is either a α-pyrone (flavonols and flavanones) or its dihydroderivative (flavonols and flavanones). According to the position of the benzenoid substituent flavonoids are separated into flavonoids (2-position) and isoflavonoids (3-position). Flavonols, the most common flavonoids in food, vary from flavanones based on hydroxyl group at the 3-position and a C2–C3 double bond. Flavonoids are usually hydroxylated in positions 3,5,7,2,3′,4′ and 5′ (Ayaz et al., 2019). The most investigated flavonol compounds are rutin and quercetin, and they are mainly present in buckwheat, asparagus, and citrus fruits, as well as in peaches, apples, and green tea. In relation to flavones (e.g., apigenin, luteolin) as the greatest sources of this subclass stand out celery, red peppers, chamomile, mint, parsley, rosemary, oregano, and ginkgo biloba (Truzzi et al., 2021). Grapes and berries are well known sources of flavanols where they are present in the form of (+)-catechin and (−)-epicatechin (Gadkari and Balaraman, 2015). Isoflavones, particularly daidzein, dominantly exist in leguminous plants, and they can be found in large quantities in soybeans and soymilk (Truzzi et al., 2021). Widely-known flavanones including naringin, neoeriodictyol and neohesperidin are usually found in citrus fruits and their products. Other flavanones are present in some medicinal plants such as mentha (Nazzaro et al., 2020). Dietary sources of anthocyanins (e.g., cyandin, malvidin) involve different red and purple berries, grapes, as well as apples, plums, cabbage, and other types of foods comprising high level of natural colorants (Mattioli et al., 2020).

Flavonoids exist in various forms such as aglycones, glycosides and their methylated derivatives. In case of glycosides, the glycosidic bond is ordinarily placed at positions 3 or 7 and the carbohydrate can be L-rhamnose, D-glucose, glucorhamnose, galactose, or arabinose. Methyl ethers and acetyl esters of the alcohol group are also identified in nature (Kumar and Pandey, 2013).

## Non-flavonoid compounds

### Tannins

Tannins is a group of naturally occurring high molecular weight polyphenols with a relatively complex structure. In nature they can be found in complexes with alkaloids, polysaccharides, and proteins and further divided into hydrolysable tannins and condensed tannins (also known as proanthocyanidins). Polymers of catechin, epicatechin, and/or leucoanthocyanidin, commonly named condensed tannins, are the most abundant polyphenols in woody plants. The degree of polymerization differs significantly and can range from several to more than 50 flavanol molecules. While small molecules of condensed tannins are soluble in aqueous or organic solvents, the large polymers are insoluble, and this disturbs their analysis, comprising determination of their content in food (Khanbabaee and Van Ree, 2001). Esters of gallic and ellagic acids are termed hydrolysable tannins. The center of the hydrolysable tannin molecule represents a glucose ring and its hydroxyl group can be esterified with gallic or ellagic acid, obtaining gallotannins or ellagitannins, respectively (Singla et al., 2019).

### Phenolic acids

Polyphenols with a carboxylic acid are called phenolic acids. They are abundant in plant-based foods with highest content in seeds, fruits peel and vegetables leaves. Generally, they can be found in bounded form such, in particular as amides, esters, or glycosides and sparsely as free (Kumar and Goel, 2019). There are two representative parent structures of phenolic acids. In the case when the carboxyl group is directly attached to the phenol ring, the phenolic compound is known as hydroxybenzoic acid. In the case when C=C bond separated the carboxyl group and phenolic ring, these molecules are named hydroxycinnamic acids (Al Mamari, 2021). Substituted derivatives of hydroxybenzoic and hydroxycinnamic acids are the principal phenolic acids in plants, and hydroxycinnamic acids are with greater incidence. Phenolic acids can varying in the pattern of the hydroxylation and methoxylation in their aromatic rings. The most usual hydroxycinnamic acids are caffeic, *p*-coumaric and ferulic acids. They are often present in foods as simple esters with quinic acid or glucose. Perheps the most well-known bound hydroxycinnamic acid is chlorogenic acid, which provide combination of caffeic and quinic acids. Diverse from hydroxycinnamates, hydroxybenzoic acid derivatives are generally found in foods in the form of glucosides and molecules as *p*-hydroxybenzoic, vanillic and protocatechuic acids are the most common forms (Shahidi and Ambigaipalan, 2015).

### Lignans

Lignans are a non-flavonoid compounds characterized with two propylbenzene units (C6-C3) linked together between the β-position in C8 of the propane side chains. Positions C9 and C9ˋ are substituted in various arrengements, having as a result in a high diversity of their structural forms. Accordingly, lignans are organized in eight subgroups: furans, furofurans, dibenzylbutanes, dibenzylbutyrolactones, dibenzocyclooctadienes, dibenzylbutyrolactols, aryltetralins and arylnaphthalenes. Lignans are phytoestrogens and can be found in legumes, seeds, and vegetable oils. In high amounts they can be present in flaxseed and flaxseed oil, with the prominent compounds being secoisolariciresinol and matairesinol (Amawi et al., 2017). Lignans are generally present in their free forms, whereas glycosylated molecules are not abundant (Ayres and Loike, 1990).

### Stilbens

Another comparably minor class of non-flavonoids is stilbenes (1,2-diarylethenes) which are characterized by two phenyl moieties connected together by a two-carbon methylene groups. In stilbenes, the m-positions in ring A are usually substituted by two hydroxyl groups, while different positions in ring B may be substituted by hydroxyl and methoxyl groups. Stilbenes occur as cis and trans isomers, and they can exist in free (minor) and glycosylated (major) forms. Stilbenes are not usual in plants and they are generated just upon pathogen invasion (Amawi et al., 2017). One of the most analysed stilbenes is resveratrol (3,5,4′- trihydroxystilbene), that is produced by several plant species, in particular grapes, peanuts, and berries (Shen et al., 2009). Generally, the red wine is accepted as source for stilbenes, and those are resveratrol, piceid, piceatannol, astringin and pterostilbene and their dimers (Zhang et al., 2021). Also, some plant species from *Polygonum* genus, like *Polygonum cuspidatum*, which have not been applied as dietary ingredients, contain high content of stilbenes (Nonaka et al., 1982).

## Nanoformulations of plant polyphenols used in depression

Nanotechnology and bio-inspired nanoparticles have got great attention these days and are extensively reported in pre-clinical studies against various diseases (Ovais et al., 2018a; Qasim Nasar et al., 2019; Chittaranjan Patra et al., 2021; Sani et al., 2021). The application of non-targeted antidepressants can provoke various side effects besides their low efficacy (Haque et al., 2014). One of the strategies in treatment of depression is to develop nanotechnology-based drug delivery systems. Nano-formulations have numerous advantages since they have specific control, as well as continued and targeted release characteristics (Ovais et al., 2018c; Khalil et al., 2018; Ovais et al., 2019; Ayaz et al., 2020; Nasar et al., 2022).

### Curcumin

Antidepressant activity of curcumin has been tested in various animal models, and it was shown that it acts through decreasing inflammation, ameliorating oxidative stress induced apoptosis, and regulating the release of serotonin and dopamine. Animal and *in vitro* investigations have demonstrated that administration of curcumin could regulate the level of serotonin and dopamine in the central nervous system (Asadi et al., 2020). Main drawbacks of curcumin use as a therapeutic agent is its considerably low water solubility, insufficient permeability cross the blood-brain barrier (BBB), and poor bioavailability. These major challenges make curcumin problematic for use as an optimal antidepressant agent. Researchers utilized solid lipid nanoparticles (SLNs) to encapsulate curcumin (Cur) together with dexanabinol (HU-211). HU-211 represents an artificially synthesized cannabinoid derivative without cannabimimetic effects. Due to its highly lipophilic nature, HU-211 may present a new treatment procedure for MDD. On the contrary to curcumin, HU-211 use is interrupted by its low stability in biological systems and poor cellular uptake. To overreach these limitations, nanotechnology-based drug delivery systems are prospective strategy. The antidepressant effects of the dual-drug nanoparticles (Cur/SLNs-HU-211) for MDD treatment were analyzed in corticosterone-induced cellular and animal models of MDD. According to the results, Cur/SLNs-HU-211 effectively protected PC12 cells from corticosterone-induced apoptosis and induced higher dopamine level release, which may be connected with the higher uptake of Cur/SLNs-HU-211 exhibited by cellular uptake behavior analysis. Moreover, Cur/SLNs-HU-211 nanoparticles significantly decreased the immobility time in forced swimming test, improved fall latency in rotarod test, and increased the dopamine level in mice blood. Cur/SLNs-HU-211 can deliver more Cur to the brain providing that way a significant enhancement in neurotransmitters level in brain tissue, primarily in the hippocampus and striatum. The data obtained using Western blot and immunofluorescence demonstrated that Cur/SLNs-HU-211 nanoparticles can significantly improve the expression of CB1, p-MEK1, and p-ERK1/2.

Also, Asadi et al. (2020) performed a double-blind, randomized clinical trial designed to examine impact of nano-curcumin supplement on depression, anxiety and stress level in diabetic patients with peripheral neuropathy (Asadi et al., 2020). Eighty patients entered the study and the participants were divided randomly in two groups: intervention and control groups. They received 80 mg of nano−curcumin or placebo capsules per day during 8 weeks. At baseline of the investigation, as well after it, anthropometric measurements, dietary intake, physical activity, glycemic indices, and severity of neuropathy were evaluated. Depression, anxiety, stress scale questionnaire was used for measuring of the depression, anxiety, and stress level before and at the end of the intervention. According to the obtained data, there was a significant reduction in the mean score of depression in the nano-curcumin group after the study completion (from 16.7 [3.1] to 15.3 [2.6]) in comparison to placebo group (17.5 [3.2] to 17.3 [3.1]). These outcomes implied that application of nano-formulation with curcumin during 8 weeks was beneficial in alleviating depression.

### Baicalein

Chen et al. (2018) analysed anti-depressant effect of baicalein-loaded solid lipid nanoparticles in animal model. These particles were modified using N-acet Pro-Gly-Pro (PGP) peptide aiming to promote binding of nanoparticles to neutrophiles *in vivo* and achieve brain-targeted delivery (Chen et al., 2018). In previous study, Lee at al. (2013) showed both acute and chronic anti-depressant effects of baicalein (Lee et al., 2013). After i.p application 1–4 mg kg^−1^ of baicalein the immobility time was significantly lowered in forced swimming test and tail suspending test. As stated by the same group of authors, chronic administration of equal baicalein doses during 21 days decreased the immobility time and enhanced locomotor activity in chronic unpredictable mild stress model in rats. Higher doses (10–40 mg kg^−1^) applied i.p. 30 min prior to daily exposure to continual restrained stress reduced depression-like behavior 14 days afterwards the application. According to Chen et al. (2018) brain distribution assay showed that baicalein-loaded solid lipid nanoparticles modified using PGP enhanced drug concentration in BLA region greatly (Chen et al., 2018). This region is the major one connected with emotional and psychiatric disturbances. Results of the study also revealed that the tested nanoparticles managed to lower immobility time, improve swimming and climbing time and weaken locomotion in olfactory-bulbectomized rats.

### Silymarin

Ashraf et al. (2019) investigated the effect of silymarin alone or its nanostructured lipid carrier formulation on depressive-like behavior triggered by chronic unpredictable mild stress (CUMS) (Ashraf et al., 2019). During this research mice were exposed to CUMS pattern for 14 days. Within 2 weeks animals received silymarin (200 mg kg^−1^, p.o.) by itself or its nanoparticle formulation, or fluoxetine (10 mg kg^−1^, p.o.). On the 15th day behavioral and biochemical parameters were analyzed. According to the results of behavioral despair tests, oral application of silymarin (200 mg kg^−1^), especially in the form of nanoparticles, demonstrated an antidepressant-like effect that was comparable with the one showed by fluoxetine. Silymarin nanoparticles achieved reduction of weight gain, increased immobility time in both the teil suspension test and forced swimming test, in addition to the decreased time spent grooming in the splash test. In the same study silymarin reversed prefrontal cortical and hippocampal oxidative stress and neuroinflammation induced by CUMS. Additionally, it increased neurotransmitter levels (NE and 5-HT levels), strengthened neurogenesis and suppressed the activation of nod-like receptor protein 3 inflammasome. For certain parameters, nanoparticles of silymarin exhibited higher effect compared to silymarin most likely as a result of significantly higher brain concentration of silybinin that represent the principal active component of silymarin. Namely, its concentration was almost 12-fold higher in the group that received silymarin nanoparticles in comparison to the group treated with silymarin alone. The authors concluded that antidepressant-like activity of silymarin might be assigned to its antioxidant and anti-inflammatory effects together with enhanced neurogenesis in the prefrontal cortex and hippocampus.

### Gallic acid

Chhillar and Dhingra (2013) demonstrated recently anti-depressant effect of gallic acid in mice subjected to unpredictable chronic mild stress (Chhillar and Dhingra, 2013). Similarly, Nabavi et al. (2016) showed antidepressant-like activity of gallic acid applied intraperitoneally in balb/c mice with post-stroke depression (Nabavi et al., 2016). Aiming to enhance its therapeutic effect, targeted delivery of gallic acid to brain is the imperative for showing an effective; better tolerated anti-depressant activity. Nagpal et al. (2012) investigated the anti-depressant activity of gallic acid loaded chitosan nanoparticles (GANP) and accordant tween 80 coated batch (cGANP) in mice (Nagpal et al., 2012). Both types of nanoparticles were applied in a quantity equivalent to 10 mg kg^−1^ gallic acid once per day during 1 week. Anti-depressant activity was evaluated using Despair Swim Test and Tail Suspension Test. Based on the significant enhancement of *in vivo* pharmacodynamic activity; better MAO-A inhibition; and stronger *in vivo* antioxidant activity obtained using cGANP authors presented the favorable outcome of ligand coated nanoparticulate system for the delivery of gallic acid across brain.

### Quercetin

Bhutada et al. (2010) showed the promising role of quercetin in depressive-like disorders (Bhutada et al., 2010). It improved behavioral disorders in mice and rats with anxiety and depressive-like behaviors provoked with the corticotropin-releasing factor. Nevertheless, low absorption, fast metabolism and narrow capacity for crossing the blood-brain-barrier are restraints for its application in the treatment of neuropsychological diseases. Due to excessive first-pass metabolism, the effective dose of quercetin required to achieve the neurological activities might be considerably high. Nasal utilization provides various advantages such as avoiding the hepatic presystemic metabolism, simple dose regulation, continuous absorption and great convenience of application in patients (Xu et al., 2020). Also, nasal drug delivery bypasses the blood-brain barrier; it targets drugs directly to brain across neural connections between the olfactory epithelium, olfactory bulb, trigeminal nerve, and finally, the brain (Trevino et al., 2020). Tong-Un et al. (2010) determined the anti-depression like activity of quercetin liposomes applied nasally. Male Wistar rats were received quercetin liposomes, containing 20 μg in one dose, *via* intranasal route once daily continually for 4 weeks (Tong-Un et al., 2010). Anti-depressant effect was assessed employing forced swimming test and the results exhibited that free liposomes and vehicle (PEG) treatment alone did not provide significant changes in immobility time at all treatment intervals applied in this investigation. At the same time, quercetin liposomes induced a considerable decrease in the immobility time at all treatment duration. The anti-depressant generally reduces immobility time without stimulating motor activity. According to the same group of authors, the forced swimming test data showed that the acute and the repeated administration of quercetin liposomes acted like an anti-depressant drug in rat, by decrease of the immobility time. Altogether, the obtained data proposed that the mechanism underlying the anti-depression activity of nasally applied quercetin liposomes may include the inhibition MAO-A and increase the serotonin level. Furthermore, the electropharmacogram of adult rats which received quercetin have the same pattern as the well-known antidepressant moclobemide, MAO-A inhibitor (Dimpfel, 2009).

## Mechanisms underlying the anti-depressant effects of phytochemicals

### Efficacy of poly-phenols in depression

**FIGURE 3:**
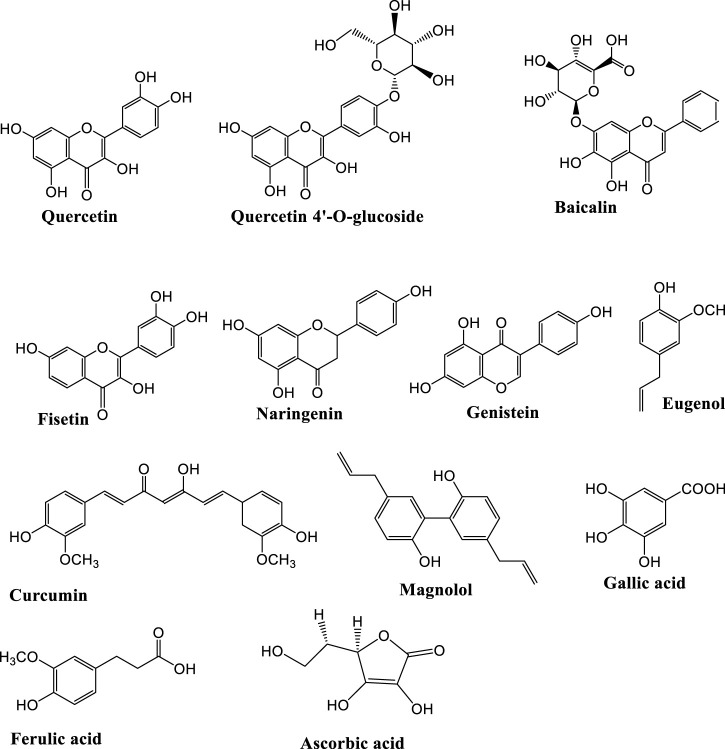
Chemical structures of important anti-depressant flavonoids.

**FIGURE 4:**
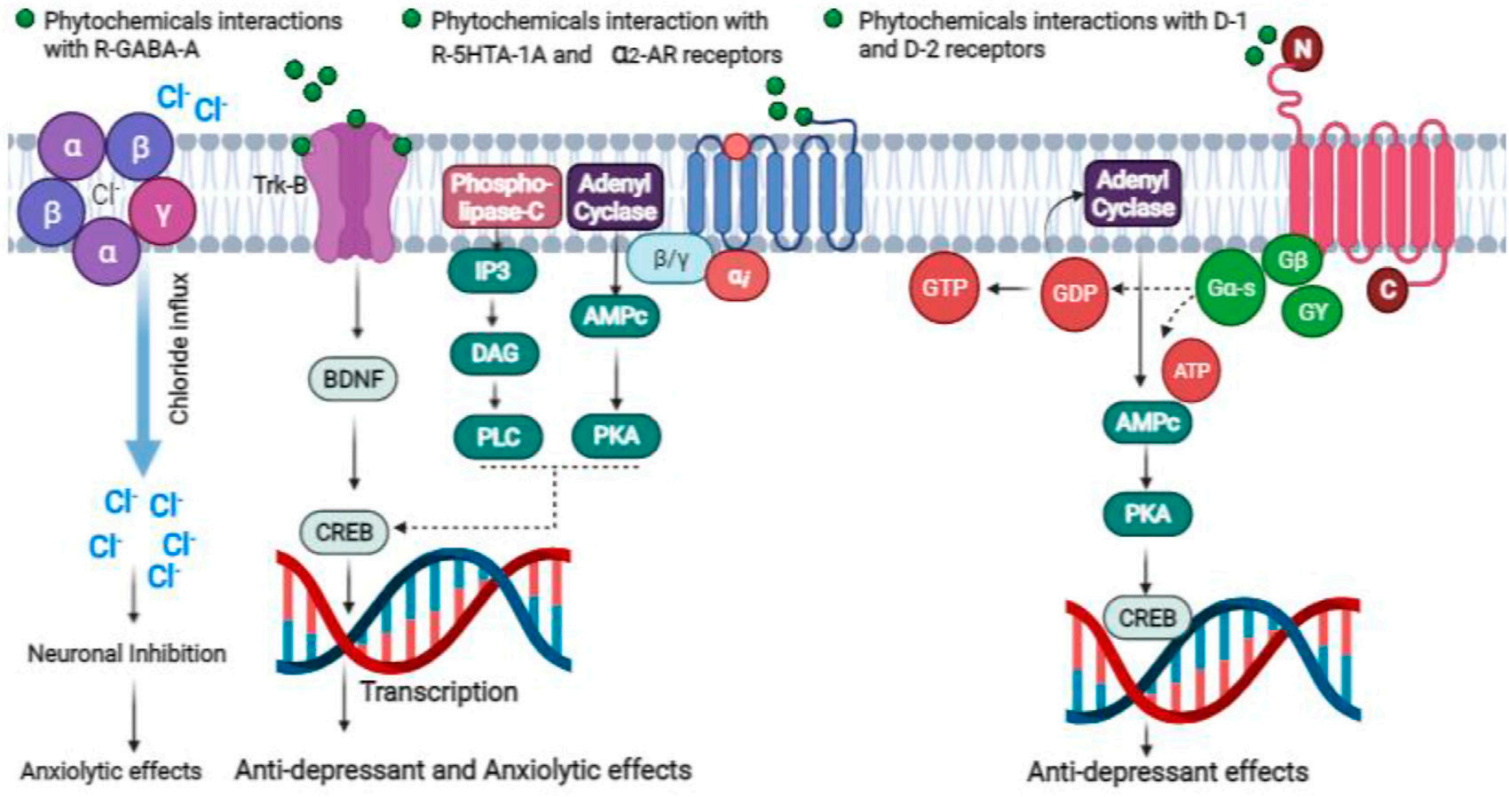
Mechanisms underlying the anti-depressant and anxiolytic effects of phytochemicals.

The subgroups of phenolic compounds includes flavonoids, coumarins, chromones, antraquinones, phenolic acids (carboxylic acid derivatives), lignanes, and stilbenes (Garcia-Salas et al., 2010; Ayaz et al., 2017b) (Figure 2). Flavonoids are phenolic compounds whose antiviral, antibacterial, hepatoprotective, antioxidant and anti-inflammatory activities have also been reported (Kumar and Pandey, 2013). They have been widely investigated for their antidepressant effects (Figures 3, 4; Table 1). Genistein is an isoflavone that can cross blood brain barrier (BBB) in mice (Si and Liu, 2008) and rats (Tsai, 2005) and its antidepressant effects on chronic use for 21 days, have been reported in male mice on forced swimming test (FST) and tail suspension test (TST) (Hu et al., 2017). In one study, the antidepressant effects of quercetin 4′-O-glucoside or quercetin has been reported in mice on FST and it was demonstrated that quercetin 4′-O-glucoside at a dose of 20 mg kg^−1^ produced the same effects as produced by fluoxetine at 20 mg kg^−1^ (Singh et al., 2021). Another study showed that quercetin administration at 10 mg kg^−1^ for 14 days decreased immobility time on TST but did not reduce immobility time on FST, however it was further found immobility time was reduced on both tests (TST and FST) at 25 and 50 mg kg^−1^ (Holzmann et al., 2015). The antidepressant mechanism of quercetin may be due to NMDA receptors inhibition that result in reduced intracellular calcium level which further leads to inhibition of protein calmodulin and then neuronal nitric oxide synthase resulting in decreased nitric oxide levels (NO) (Holzmann et al., 2015). In the brain, glutamate is the main excitatory neurotransmitter and its increased level in the synapse causes excessive stimulation of N-methyl-D-aspartate (NMDA) receptors that result in NMDA-mediated excitotoxicity and various forms of damage, such as a massive influx of calcium and the release of nitric oxide (NO) (Bishop and Anderson, 2005). Increase production of nitric oxide (NO) leading to further glutamate release causing further oxidative stress and excitotoxicity. Additionally, several studies have reported that nitric oxide synthase inhibition can cause antidepressant-like effect (Zeni et al., 2011).

**TABLE 1:** Details of Phytochemicals, their sources and anti-depressant effects.

Bioactive compounds	Experimental design	Results/Mechanisms of anti-depressant effects	References
Genistein	Tail suspension and Force swimming tests (FST) in male ICR mice	↑Anti-depressant effects	Hu et al. (2017)
		↑the anti-depressants effects of 5-HT_1A_ agonist (O-OH-DPAT)	
Quercetin	Force swimming test and Tail suspension test in mice model	↑ Anti-depressant effects	Holzmann et al. (2015)
		↓Lipid hydro-peroxide contents	
		↔Nitric oxide and glutamate	
Quercetin 4-O-glucoside	Force swimming test in mice model	↑Anti-depressant effects	Singh et al. (2021)
		↓MAO-B	
		↑Brain 5-HT level	
		↑Anti-radicals effect	
Hesperidin	Tail suspension test in mice model	↑ Anti-depressant effects	Donato et al. (2014)
		↑BDNF levels in the hippocampus	
Kaempferitrin	Force swimming and tail suspension tests in mice model	↓Depression	Cassani et al. (2014)
		↑5-HT synthesis	
		↓5-HT_1A_ receptors	
Baicalin	Sucrose preference test in ICR mice	↓Depression	Zhang K. et al. (2019)
		↑Neurogenesis *via* Akt/FOXG1 pathway	
Fisetin	Force swimming and tail suspension tests in ICR mice	↓Depression	Wang et al. (2017)
		↑Trk-B signaling pathway	
Naringin	Forced swimming test in mice model	↓Depression	Ben-Azu et al. (2019)
		↑Anti-radicals, cholinergic pathways	
		↓NO, MDA and reduce inflammation	
β-sitosterol	Forced swimming and tail suspension tests in Kun-Ming mice	↓Depression	Zhao et al. (2016)
		↑5-HIAA	
		↑Brain 5-HT and Norepinephrine (NE)	
α and β-amyrin	Forced swimming test in Male mice	↑Anti-depressant effects	Aragão et al. (2006)
Linalool and (-)-linalool	Tail suspension and forced swimming tests	↓ Depression	Coelho et al. (2013); Guzmán-Gutiérrez et al. (2015)
		↑Brain 5-HT and Norepinephrine (NE)	
Rosmanol	Tail suspension and forced swimming tests	↑Anti-depressant effects	Abdelhalim et al. (2015)
Oleanolic acid and Ursolic acid	Tail suspension and forced swimming tests	↑Α_1_β_2_ϒ_2_L GABA-A receptors	Colla et al. (2021)
		↑Anti-depressant effects	
Ursolic acid	Tail suspension and forced swimming tests	↑5-HT synthesis	Machado et al. (2012), Colla et al. (2015)
		↓5-HT_1A_ activation	
		↑D_1_ and D_2_ receptors	
Betulinic acid and carnosol	Tail suspension test in male mice	↑Anti-depressant effects	Machado et al. (2013)
Evodiamine	Forced swimming and sucrose preference tests in rats	↑Anti-depressant effects using CUMS model	Jiang et al. (2015)
Β-pinene	Force swimming test in ICR mice	↑D1 and β-adreno-receptors	Guzmán-Gutiérrez et al. (2015)
		↓5-HT_1A_	
		↓Depression	
Α-spinasterol	Force swimming test	↑TRPV1 antagonism	Socała and Wlaź (2016)
		↓Depression	
Fucosterol	Tail immersion and force swimming tests in mice	↑Brain 5HT, BDNF and nor-epinephrine levels	Zhen et al. (2015)
Protopine	Tail suspension test in mice	↓Depression symptoms	Xu et al. (2006)
Harmine	Force swimming and sucrose preference tests in CUMS rats	↓Depression symptoms	Fortunato et al. (2010a)
Mitragynine	Force swimming and tail suspension tests in male mice	↔neuro-endocrine axis HPA	Idayu et al. (2011)
		↓Depression	

Key: ↑: Activate, Augment, Synergize, ↓:Reduce, down-regulate, suppress ↔: Modulate, 5-HT_1A_: 5-hydroxytryptamine _1A_, MAO-B, Monoamine oxidase B; D1, Dopamine1; D2, Dopamine2; NO, nitric oxide; MDA, malondialdehyde; TRPV1, Transient receptor potential vanilloid 1; HPA, hypothalamic pituitary adrenal; CUMS, chronic unpredictable mild stress.

Another study explored the antidepressant potential of chrysin at 20 mg kg^−1^ for 14 days in male OB C57B/6J mice using splash test (SP), where antidepressant effects were measured as increased in gromming time at 5 and 20 mg kg^−1^ doses decreased immobility time in obese (OB) mice on FST, but the hippocampal concentration of 5-HT and brain-derived neurotrophic factor (BDNF) was found increased (Borges Filho et al., 2016).

In another study, fisetin administration at 5 mg kg^−1^ increased the activation of the tropomyosin kinase B receptor (TrkB) through its phosphorylation in the hippocampus, which may result in pro-neurogenesis (Wang et al., 2017) reflecting its antidepressant effect on TST and FST. Apart from this fisten reversed depression in mice induced by spatial restraint stress which was demonstrated on FST and TST (Wang et al., 2017). Other studies have shown that dihydromyricetin triggered the ERK1/2-CREB pathway and enhanced glycogen synthase kinase-3 beta (GSK-3 beta) phosphorylation at ser-9, resulting in elevated BDNF expression in the hippocampus while suppressing neuroinflammation. These results might be explained by the antidepressant effect seen on TST and FST after administration of 10 and 20 mg kg^−1^ for 3 days (Ren et al., 2018). Hesperidin is another flavonoid, which may raise hippocampal BDNF levels at 0.3 and 1 mg kg^−1^ daily dose for 21 days. Hesperidin produced antidepressant effect at 0.3 and 1 mg kg^−1^ on TST was found similar to the effects produced by imipramine at 15 mg/kg and fluoxetine at 32 mg kg^−1^ (García-Ríos et al., 2020).

Baicalin is another flavonoid that has been proven to have CNS effects. It may enhance neuronal differentiation by increasing BDNF and extracellular signal-regulated kinase (ERK) phosphorylation (Xiong et al., 2011). The glucuronide glycoside of baicalin showed antidepressant activity by reducing monoamine oxidase (MAO)-A and B enzymes (Zhu et al., 2006). It has been suggested that baicalin at a 60 mg kg^−1^ produced the same effect on TST and Standard Penetration Test (SPT) as that produced by fluoxetine at 15 mg kg^−1^ (Zhang R. et al., 2019). Another flavonoid with antidepressant and antioxidant properties is naringin, which decreased immobility on the FST when administered at a dose of 2.5, 5, and 10 mg kg^−1^ for 7 days. The antidepressant effect of these doses was associated with increased cholinergic transmission as a result of decreased activity of the enzyme acetylcholinesterase and antioxidant defence systems caused by increased GSH levels, as well as increased activity of superoxide dismutase (SOD) and catalase (CAT) in mice brains.

Apart from this, naringin decrease ROS level and nitrogen species resulting in inhibition of nitrosative processes and lipid peroxidation (Ben-Azu et al., 2019). Naringenin is a major flavonoid of grapefruit, whose antidepressant activity has been reported, it increase NE, 5-HT, and BDNF levels and glucocorticoid receptors (Yi et al., 2014). Luteolin is a major flavonoid of the *Cirsium japonicum* extract, decreased the immobility time on FST at 5 and 10 mg kg^−1^ dose in the same manner as decreased by the antidepressant imipramine at a dose of 5 mg kg^−1^ (De La Peña et al., 2014).

### Antidepressant effects of other phenolic compounds

Apart from flavonoids, other phenolic compounds are also reported for their antidepressant effects. Bis-eugenol showed antidepressant effect due to synthesis of 5-HT and participation of dopamine (DA) receptors (do Amaral et al., 2013). In depression models, magnolol from *Magnolia officinalis* bark altered brain BDNF level as well as serotonergic, noradrenergic and dopaminergic neurotransmission (Li et al., 2013). Among coumarins related compounds, scopoletin by affecting DA D1, D2, and 5-HT2a receptors and α1 and α2 adrenoceptors and psoralen by altering HPA axis demonstrated antidepressant effect in animal depression models (Capra et al., 2010).

Among the phenolic acids related compounds, the antidepressant like effects of paeoniflorin, galic acids, eugenol, freulic acid and ascorbic acid have been reported previously. Paeoniflorin from *Paeonia lactiflora* root (ancient Chinese antidepressant) demonstrated antidepressant effect by affecting HPA axis and by up-regulation of serotonergic system (Qiu et al., 2013). Gallic acid decreased plasma nitrite, corticosterone and malondialdehyde levels as well as prevented MAO-A activity induced by stress (Chhillar and Dhingra, 2013). Eugenol showed antidepressant like effects by increasing hippocampal BDNF level and inhibiting MAO-A and MAO-B effects (Irie, 2006). Ferulic acid showed antidepressant effects by interaction with 5-HT1a and 5-HT2a receptors (Zeni et al., 2012). Another phenolic acid is ascorbic acid (vitamin C), demonstrated antidepressant effects by serotonergic, dopaminergic and noradrenergic systems, NMDA receptors, K^+^ channels, alteration of mammalian target of rapamycin pathway and l-arginine-nitric oxide-cyclic guanosine monophosphate (l-arginine-NO-cGMP) pathway (Moretti et al., 2014). Another phenolic compound is curcumin, showed antidepressant effects by elevation of BDNF and brain monoamine levels, reduction of pro-inflammatory cytokines and prevention of MAO-A and MAO-B activities (Jiang et al., 2013).

### Alkaloids as potential anti-depressant agents

**FIGURE 5:**
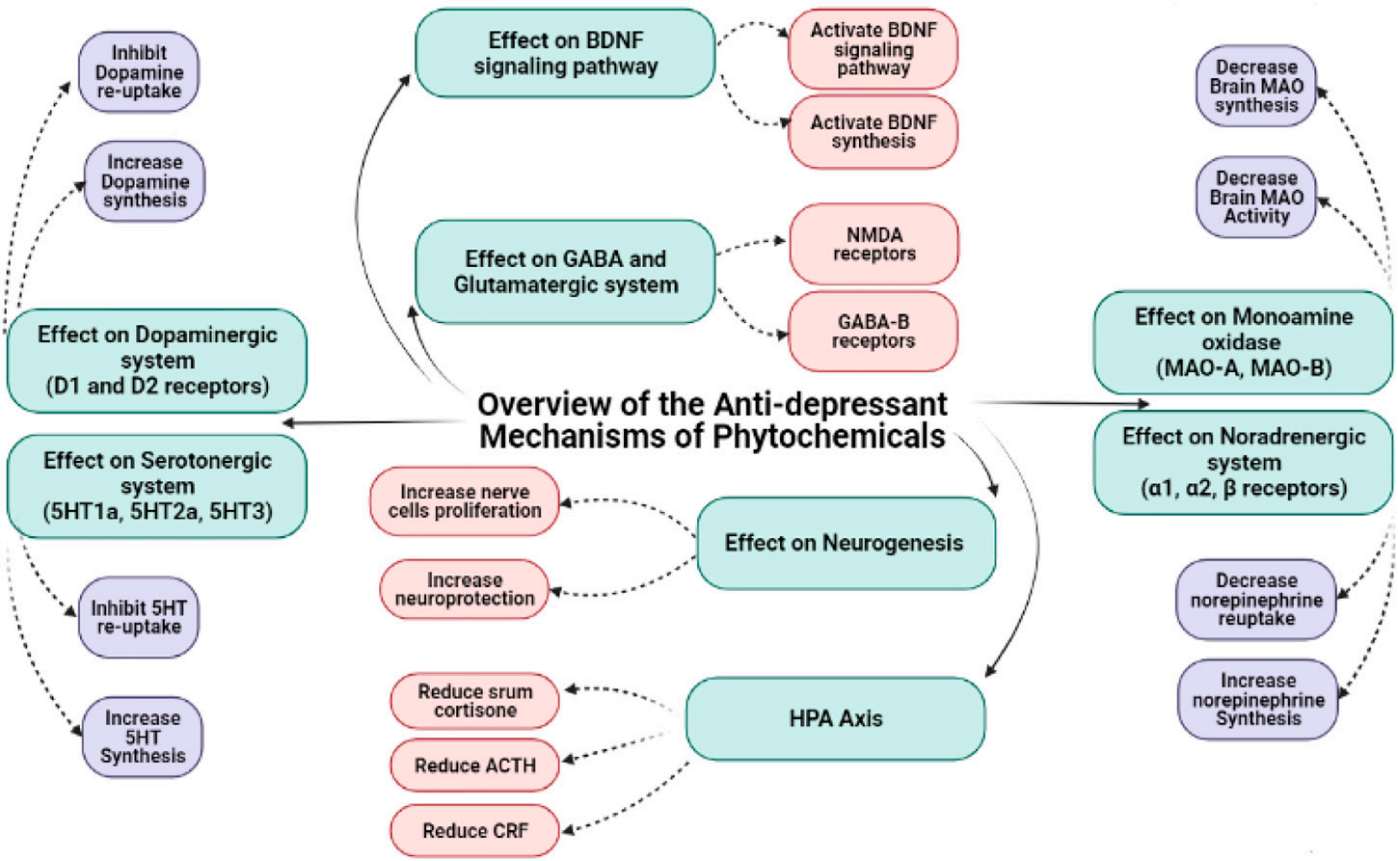
Overview of the anti-depressant mechanisms of phytochemicals.

Alkaloids are nitrogenous pharmacologically active secondary plant metabolites having diverse chemical structures and obtained from crude acid-base extracts (Perviz et al., 2016) (Figure 5). The anti-inflammatory tranquilizer and antiarthritic potential of isoquinoline alkaloids, morphine and colchicine respectively have been demonstrated previously (Lopes et al., 2018). Using FST in rats, berberine at a dose of 50 mg kg^−1^, increases climbing behavior and decreases immobility which reflects antidepressants effects (Lee et al., 2012). The antidepressants effects of *Annona cherimola* extract containing number of alkaloids including liriodenine, anonaine, nornuciferine and 1,2-dimethoxy-5,6,6a,7-tetrahydro-4H-dibenzoquinoline-3,8,9,10-tetraol, have been reported in mice FST. These alkaloids have been suggested to increase 5-HT and DA (Martínez-Vázquez et al., 2012).

The dose dependent antidepressant effects of β-carbolines derivatives such as harmane, norharmane and harmine have been reported previously (Farzin and Mansouri, 2006). The antidepressants effects of harmine has been compared with imipramine at different doses for 14 days in rats using FST, BDNF protein levels were increased by harmine in the hippocampus of rats while imipramine did not increase BDNF protein levels, reflecting that antidepressant effect is due to rise in hippocampal BDNF level (Fortunato et al., 2010a; b). One study reported that harman infusion increases 5-HT concentration and decreases degradation of 5-HT metabolite levels due to MAO-A inhibition (Baum et al., 1996). Another study suggested that harmane injection increase plasma concentration of corticosterone, adrenocorticotrophic hormone (ACTH), noradrenaline (NA) and 5-hydroxytryptamine (5-HT) in the structures of limbic system, suggesting that harman can modulate brain neurochemistry, behavioral alterations and neuroendocrine functions by inhibiting MAO-A (Smith et al., 2013).

The alkaloid fractions of *Rhazya stricta* extract containing rhaziminine, akuammidine, and tetrahydrosecamine inhibited MAO-B enzyme after oral administration to male rats for 21 days, which is responsible for antidepressant-like effect (Ali et al., 1998). Napelline, hypaconitine, songorine, and mesaconitine are diterpene alkaloids of *Aconitum baicalense* which have shown antidepressant effects in animals depression model by improving the activity serotonergic system (Perviz et al., 2016).

Mitragynine is *Mitragyna speciosa* alkaloid, whose antidepressant effects have been shown on TST and FST in mice, where it reduced immobility time (Idayu et al., 2011). Administration of punarnavine for 14 days at a dose of 20 and 40 mg kg^−1^ resulted in decrease corticosterone levels, MAO-A activity and immobility on FST in both unstressed and stressed mice (Dhingra and Valecha, 2014). Apart from this, piperine on FST and protopine on TST showed antidepressant effects which may be due serotonergic mechanism (Wattanathorn et al., 2008) and 5-HT inhibition respectively (Xu et al., 2016). The antidepressant potentials of alkaloids have been clearly demonstrated in various preclinical studies but more studies are required to further evaluate their efficacy, potency and safety.

### Antidepressant effects of saponins and sapogenins

Saponins are secondary metabolites of plants and mostly found in glycosilated forms (Ayaz et al., 2016). Saponins contain one to three sugar chains which may be straight or branched and usually composed of d-glucose, d-galactose, d-glucuronic acid, l-rhamnose, l-arabinose, d -fucose or d-xylose (Vincken et al., 2007). Sapogenins are generally classified into triterpenoidal (with 4–5 rings skeleton) and steroidal (with 5–6 rings backbone) groups. The antidepressant effect of bacopaside I from *Bacopa monniera* in various animal models has reported (Liu et al., 2013). Ginsenosides from ginseng and intestinal metabolite of ginseng, 20(S)-protopanaxadiol demonstrated antidepressant effects by enhancing brain monoamine levels, HPA axis, BDNF signaling pathway and hippocampal neurogenesis (Xu et al., 2010). Glycyrrhizin is a triterpene saponin, exhibit antidepressant effect due to participation of α1 adrenoceptor and DA D2 receptor (Dhingra and Sharma, 2005). Hederagenin of *Akebia quinata*, showed antidepressant effect by HPA axis modulation (Jin et al., 2012). Sarsasapogenin from *Anemarrhena asphodeloides* possess antidepressant activity by preventing MAO-A and MAO-B activities and increasing NE and 5-HT levels of the hippocampus and hypothalamus (Ren et al., 2006).

### Phyto-sterols in depression

**FIGURE 6:**
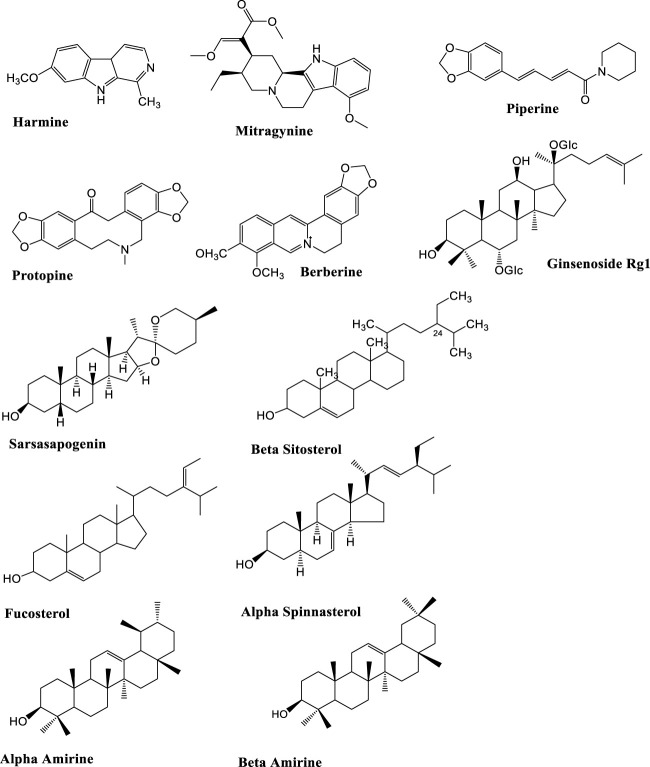
Chemical structures of potential anti-depressant alkaloids, saponins and sterols.

The chemical structure of plant sterols, also called phytosterols is similar to the structure of cholesterol and mostly found in nuts, cereals, fruits and vegetables (Ayaz et al., 2017a; Shah et al., 2019) (Figure 6). β-sitosterol, stigmasterol fucosterol, and campesterol are among the different identified types of sterols. Sterols can cross blood brain barrier (BBB) to exert their effects on CNS due to their glycosylated forms and lipidic nature (Tabata et al., 2008). Trevisan et al. suggested that α-spinasterol exhibits antagonistic effects on transient potential receptors V1 (TRPV1). The activation of these receptors in various parts of brain may increase the release of glutamate, GABA, or other catecholamines (Martins et al., 2014), underlying the mechanism of anxiety and depression due to involvement of TRPV1. The antidepressant effect of α-spinasterol in male mice subjected to FST, was verified by Socała and Wlaź (2016). Socała and Wlaź further suggested that α-spinasterol may activate CB1 receptors which in turn activate TRPV1 receptors simultaneously to inhibit their anxiolytic effects (Socała and Wlaź, 2016).

One study reported that administration of fucosterol (20 and 30 mg kg^−1^) produced antidepressant effects in male mice FST and TST similar to that of fluoxetine at a dose of 20 mg kg^−1^ (Zhen et al., 2015). In mice brains, fucosterol prevented the reduction in the levels of NA, 5-HT and 5-HTIIA caused by stress of FST, suggesting that the antidepressant effects are due to increased monoamines and decreased metabolism rate of 5-HT. Administration of β-sitosterol (30 mg kg^−1^) produced antidepressant effects in male mice on FST and TST similar to that of fluoxetine at a dose of 20 mg kg^−1^ (García-Ríos et al., 2020). Another study reported the antidepressants potential of α- and β-amyrin (αβAMY) isolated from the stem resin of *Protium heptaphyllum* on FST. Administration of αβAMY *via* p.o route at a dose of 2.5 and 5 mg kg^−1^ decreased immobility time, as decreased by imipramine at 30 and 10 mg kg^−1^ (García-Ríos et al., 2020).

## Clinical trials on polyphenols as potential anti-depressant agents

Number of clinical studies have reported that polyphenols rich foods or dietary polyphenols intake play a significant role in the prevention and treatment of depression (Godos et al., 2018). A recent clinical trial in elderly Japanese demonstrated that higher consumption of green tea is associated with lower prevalence of depression (Niu et al., 2009). The anti-depressive potential of saffron has been reported to be similar to that of synthetic antidepressant drugs like imipramine and fluoxetine, without side effects (Basti et al., 2007). It has been concluded in the meta-analysis review of clinical trials by Hausenblas that saffron supplementation can ameliorate symptoms of depression in adults having MDD (Hausenblas et al., 2013).

## Conclusion and future perspectives

Medicinal plants and their metabolites are being used for the management of various mental disorders. The phytochemicals are widely available, more tolerable and have fewer side effects as compared to synthetic drugs. The phytochemicals with antidepressant activity include rosmanol, ursolic acid, oleanolic acid, linalool, carnosol, quercetin, fisetin, naringenin, baicalin, genistein, harmine, mitragynine, piperine, protopine, beta sitosterol, fucosterol, alpha spinnasterol, alpha amirine and beta amirine belonging to different chemical classes such as terpenes and terpenoids, saponins and sapogenins, sterols, alkaloids, polyphenols, amines and carbohydrates. Among them, the most studied phytochemicals in animal depression models are berberine, piperine, curcumin, naringenin, ascorbic acid, and ginsenosides. The antidepressant activity of these phytochemicals seems to be associated with various mechanisms that include activation of tyrosine hydroxylase enzymes, inhibition of alteration of MAO-A and MAO-B, alteration in brain monoamine levels and receptors, prevention of reactive oxygen species (ROS) and NO synthesis, involvement of D1, D2, 5-HT1A, 5-HT2A, GABAA receptors and α1, α2, β-adrenoceptors, CREB (cyclic adenosine monophosphate response element-binding protein), l-arginine-NO-cGMP pathway, and BDNF signaling pathway. All these mechanisms involves differentiation and inhibition of neuronal cell apoptosis and promotion of neuronal cell survival.

Numerous phytochemicals have been reported for their antidepressant activity but only few of them have subjected into clinical trials. Unfortunately, very few clinical trials have demonstrated antidepressant effects of phytochemicals including clinical study of curcumin in human and hence further randomized and placebo-controlled clinical trials are needed to confirm their antidepressant potential of these phytochemicals. Further research studies are also needed to investigate their antidepressant mechanisms and to develop cost effective formulations for the treatment of depression.

## Abbreviations

5-HT, 5-hydroxytryptamine; *BDNF*, brain-derived neurotrophic factor; CUMS, chronic unpredictable mild stress; Cur, curcumin; DASS, Depression, Anxiety, Stress Scale; ER, endoplasmic reticulum; FDA, U.S. Food and Drug Administration; SERT, serotonin transporters; FST, forced swimming test; GANP, gallic acid loaded chitosan nanoparticles; MAO, monoamine oxidase; MDD, major depressive disorder; MEM, mitochondrial membrane; NET, norepinephrine reuptake transporters; NMDA, N-methyl D-aspartate; NO, nitric oxide; SERT, serotonin reuptake transporters; SLNs, solid lipid nanoparticles; SNP, single nucleotide polymorphism; SNRIs, Serotonin and norepinephrine reuptake inhibitors; SPT, Standard Penetration Test; SSRIs, Selective serotonin reuptake inhibitors; TCAs, tricyclic antidepressants; TrkB, tropomyosin kinase B receptor; TST, tail suspension test.
